# Multi-level determinants of comprehensive palliative care delivery for breast and cervical cancer patients in Ethiopia: using the socio-ecological model as an analytical framework

**DOI:** 10.1186/s12904-026-02300-z

**Published:** 2026-08-25

**Authors:** Kalkidan Solomon Deribe, Sarona Shewaye, Nahom Solomon Habtamu, Abigiya Wondimagegnehu, Yoseph Mamo Azmera, Adamu Addissie, Eva J. Kantelhardt, Mirgissa Kaba

**Affiliations:** 1 College of Health Sciences, School of Public Health, Addis Ababa University, Addis Ababa, Ethiopia; 2 Global and Planetary Health Working Group, Institute of Medical Epidemiology, Biostatistics and Informatics, Martin Luther University Halle Wittenberg, Halle (Saale), Germany; 3 School of Public Health, Department of Public Health, Mizan-Tepi University, Mizan, Ethiopia; 4 Global Health Partnership, Addis Ababa, Ethiopia; 5 Department of Gynecology, Martin Luther University Halle Wittenberg, Halle (Saale), Germany

**Keywords:** D*eterminants*, *Palliative care*, *Breast cancer*, Cervical cancer, Socio-ecological model, *Ethiopia*

## Abstract

**Introduction:**

Comprehensive palliative care is essential for improving the quality of life of women with breast and cervical cancer in Ethiopia, yet services remain poorly organized, largely inaccessible, and concentrated in the capital. While existing studies have quantified service gaps, the multi-level determinants enabling or hindering comprehensive care remain under-explored, particularly how these factors differ between urban and rural settings.

**Objective:**

To explore the multi-level determinants of implementing comprehensive palliative care for women with breast and cervical cancer in Ethiopia.

**Methods:**

An exploratory qualitative study was conducted from January to March 2025 in urban Addis Ababa and Sidama. Using purposive sampling with maximum variation, ninety-three participants including 37 women with breast or cervical cancer, 20 caregivers, and 36 key informants were recruited. Data were collected through open ended interview guides, transcribed verbatim, and analyzed using framework analysis guided by the Socio-Ecological Model. The analysis focused on identifying determinants at individual, interpersonal, community, organizational, and policy levels. ATLAS. ti V.09 was used to analyze the data.

**Findings:**

Determinants of comprehensive palliative care access and quality emerged across five socio-ecological levels, with urban-rural variations. At the individual level, determinants included personal health literacy, assets, and financial resources. Patient resilience and health literacy enabled care, but outweighed by catastrophic out-of-pocket costs and misconceptions of cancer as a curse more pronounced in Sidama. At the interpersonal level, social support and family dynamics were critical. Supportive families enabled care, while family stigma and partner abandonment were major barriers with stigma more intense in Sidama. At the community level, social capital, community networks, and traditional structures served as determinants. Survivor-led groups, edirs, and religious leaders enabled support, yet their impact was constrained by resource deficits. Sidama relied on informal support. Cervical cancer patients experienced stigma rooted in sexually transmitted infection leading to social exclusion, whereas breast cancer patients’ stigma was linked to fears of contagion and visible disfigurement. At the organizational level, access to healthcare services, resource allocation, and organizational culture were dominant determinants. Specialized cancer centers and community based health insurance enabled access, but systemic undervaluation of comprehensive palliative care, opioid shortages, lack of trained staff, and broken referral systems were barriers, worse in Sidama. At the policy level, governance, regulations, and health system financing were the overarching determinants. A national guideline exists but lacks rural implementation guidance, and is undermined by restrictive opioid regulations and absence of palliative care in professional curricula.

**Conclusion:**

Determinants of comprehensive palliative care exist across all levels but are systematically undermined by organizational and policy barriers. Community-based organizations and traditional structures show promise but require sustainable funding. Policy reform, opioid access liberalization, curriculum integration, and decentralized community-based service models are needed to ensure equitable, and comprehensive palliative care.

**Supplementary Information:**

The online version contains supplementary material available at 10.1186/s12904-026-02300-z.

## Introduction

Palliative care is an essential approach designed to improve the quality of life of patients and their families facing life-threatening illnesses. According to the World Health Organization (WHO), palliative care involves the prevention and relief of suffering through early identification, assessment, and treatment of pain and other physical, psychosocial, and spiritual problems [1, 2]. As WHO recommended, comprehensive palliative care is not limited to end-of-life support; it should be provided alongside curative treatments from the time of diagnosis. The WHO emphasizes that palliative care is a fundamental component of universal health coverage (UHC), meaning that all people, regardless of income, disease type, or age, should have access to these essential services without financial hardship [2]. Comprehensive palliative care therefore addresses multiple dimensions of suffering including physical symptoms, psychological distress, social isolation, spiritual concerns, economic hardship, and end-of-life care needs [3].

Globally, the demand for palliative care is rising rapidly due to the increasing burden of non-communicable diseases, particularly cancer. An estimated 56.8 million people worldwide require palliative care annually, yet only 14% of those in need actually receive it [4]. The majority of this unmet need, approximately 76%, is in low- and middle-income countries (LMICs) where health systems are already overstretched [5]. In Africa, the situation is especially critical. The continent faces a dual burden of communicable and non-communicable diseases, and cancer incidence and mortality are projected to double by 2030 [6]. Despite this growing need, palliative care services in most African countries remain fragmented, underfunded, and largely focused on pain management alone, with little attention to the psychosocial, spiritual, and economic dimensions of suffering [7, 8]. Several reviews have documented that while home-based care and community-based models exist, they are often poorly integrated with formal health systems, lack essential medications such as oral morphine, and suffer from a shortage of trained providers [9, 10].

In Ethiopia, breast and cervical cancers are the most common malignancies among women, and the majority of patients are diagnosed at advanced stages when curative treatment options are limited [11]. Despite their distinct etiologies of breast cancer influenced by genetic, hormonal, and lifestyle factors, and cervical cancer driven by persistent HPV infection, both cancers share important commonalities including late-stage presentation, remarkable symptom burdens, catastrophic costs, pervasive stigma, and the need for comprehensive palliative care [9, 11]. Studying these cancers together enables a broader understanding of systemic barriers women face, while also allowing identification of cancer-specific nuances to inform tailored interventions.

The Ethiopian government has taken important steps by developing a national palliative care guideline and including palliative care in the national health agenda [12]. However, implementation remains highly inconsistent. Existing studies have documented that palliative care services are concentrated in the capital city, Addis Ababa, leaving rural populations largely underserved [13]. Health professionals lack adequate training in palliative care, and essential medications are restricted or unavailable [14]. Furthermore, most services operate without sustainable funding, relying heavily on donor support and non-governmental organizations [15].

Despite these known challenges, most previous research in Ethiopia has focused on describing the availability of services or quantifying patient needs, as seen in recent cross-sectional studies [16, 17]. What remains poorly understood are the multi-level determinants that enable or hinder the delivery of comprehensive palliative care. The determinants of health are defined as the conditions in which people are born, grow, live, work, and age, and the systems put in place to deal with illness. These determinants include factors such as income, education, social support networks, access to healthcare, and policies. Furthermore, there is limited understanding of how these determinants interact across different levels of the social ecology, from the individual patient to the national policy environment.

Therefore, the objective of this study was to explore the multi-level determinants of implementing comprehensive palliative care for women with breast and cervical cancer in Ethiopia using the Socio-Ecological Model (SEM) as an analytical framework. The SEM recognizes that health behaviors and outcomes are influenced by multiple interacting levels; individual, interpersonal, community, organizational, and policy. It particularly suited to examine how determinants at higher levels shape what is possible at lower levels [18]. Specifically, the study aimed to: (1) describe determinants (both enabling and hindering) at the individual, interpersonal, community, organizational, and policy levels; (2) examine how these determinants interact across levels to shape comprehensive palliative care delivery; (3) compare perspectives across different stakeholder groups (patients, caregivers, health workers, community leaders, policymakers); and (4) explore variations between urban (Addis Ababa) and semi-urban or rural (Sidama) settings to inform context-sensitive recommendations for policy and practice.

## Methods

### Study design

This study employed an exploratory qualitative research design informed by a constructivist epistemological approach. This design was chosen because it allows for in-depth exploration of participants’ lived experiences, perceptions, and meanings attached to palliative care delivery. Unlike quantitative designs that measure predetermined variables, this qualitative approach enabled us to capture the complexity and context-specific nature of the determinants across multiple levels of the socio-ecological system. The study is reported following the Consolidated Criteria for Reporting Qualitative Research (COREQ) guidelines to ensure comprehensive and transparent reporting (Supplementary Material_1).

### Study area and period

The study was conducted from January to March 2025 in Addis Ababa and Sidama, Ethiopia. Addis Ababa, as the capital city of Ethiopia, serves as a central hub for healthcare services in the country. It is home to Tikur Anbessa Specialized Hospital (TASH), which stands as the largest tertiary hospital in Ethiopia. TASH plays a crucial role in oncology, as it receives cancer patients referred from various regions across the nation. Sidama is one of the semi-urban regions in Ethiopia. Within this region lies the Hawassa University Comprehensive Specialized Hospital Cancer Treatment Center (HUCSH-CTC), which has been established to cater to the growing needs of cancer patients in Sidama and its surrounding areas. These two sites were purposively selected to capture both urban (Addis Ababa) and semi-urban/rural (Sidama) perspectives, enabling comparison of palliative care delivery across different geographic and resource contexts. Both Addis Ababa and Sidama have initiated home and community-based palliative care programs. These initiatives are designed to provide palliative care to patients with life-limiting illnesses, including cancer, within their own homes and communities.

### Theoretical framework

This study was guided by the Socio-Ecological Model (SEM) as an analytical framework. The SEM conceptualizes that health behaviors and outcomes are influenced by multiple, interacting levels of influence: the individual level (knowledge, attitudes, beliefs, skills, financial assets), the interpersonal level (family, friends, healthcare providers, social networks), the community level (social norms, cultural values, community organizations, traditional structures, social capital), the organizational level (health facilities, institutional policies, resources, service delivery structures), and the policy level (laws, regulations, national strategies, funding mechanisms). The SEM was chosen because it moves beyond individual-level explanations of barriers and instead examines how determinants at higher levels shape what is possible at lower levels. This framework is particularly well-suited for studying palliative care in low-resource settings where barriers are often rooted in organizational and policy failures rather than individual shortcomings [18].

### Study participant selection

The study participants were women having a medically confirmed cervical and breast cancer, their caregivers, community and religious leaders, community volunteers, health care providers, program leaders, and experts in palliative care service delivery. Purposive sampling was employed to select participants who could provide rich, relevant, and diverse information on the phenomenon of interest. The selection was done purposively based on the information obtained from the health facilities and organizations where patients received medical and palliative care services. Maximum variation sampling was applied within the purposive approach to ensure diversity across key characteristics including cancer type (breast vs. cervical), cancer stage (early vs. advanced), geographic location (urban Addis Ababa vs. semi-urban Sidama), age, educational level, and professional background of key informants. This strategy was intended to capture a wide range of perspectives and experiences, thereby enhancing the transferability of the findings.

The total sample size for this study was determined based on the principle of data saturation, defined as the point at which no new information, themes, or insights emerged from additional interviews. Saturation was achieved after conducting 93 interviews. The distribution of participants across the interview categories was as follows: for in-depth interviews (IDI), there were 37 breast and cervical cancer patients and 20 caregivers. In terms of key informant interviews (KII), the study included 15 community-level participants, such as volunteers, community representatives, social workers, and religious leaders, along with 11 health professionals, which comprised surgeons, medical doctors, oncologists, gynecologists, nurses, health extension workers, and program leaders from various organizations. Additionally, 10 healthcare administrators from federal, regional, zonal, and woreda levels were also part of the study. This diverse representation ensured a comprehensive understanding of the palliative care landscape within the context of cancer treatment in Ethiopia.

### Eligibility criteria

The study included women with confirmed breast and cervical cancer, as well as caregivers aged 18 years and above. For patients, eligibility required a confirmed diagnosis of breast or cervical cancer at any stage, being 18 years or older, and willingness to provide informed consent. Patients who were critically ill, had severe psychiatric disorders that impaired communication, or were unable to complete the interview due to acute distress were excluded. For key informant interviews (KIIs), participants were selected based on their roles in cancer service provision including palliative care. Facility level KII participants included surgeons, medical doctors, oncologists, gynecologists, nurses, health extension workers, and program leaders were with a minimum of six months of experience working in palliative care or cancer services. KII participants at community-level included religious leaders, edir leaders, community elders, and volunteers selected based on their active community leadership roles, known engagement with cancer-affected households, and ability to provide insights into community perceptions, and support mechanisms as identified through community health worker liaison and key informant mapping. KII participants at healthcare administrators level included federal, regional, zonal, and woreda level health authorities who engaged in planning and decision making on health care, and provide oversight. Inclusion of these participants ensured evidences on determinants at multiple levels is captured.

### Data collection tool and procedure

We employed an open-ended interview guide to gather the necessary information from study participants. The interview guides were developed based on the five levels of the Socio-ecological Model and were reviewed by a panel of three palliative care experts for content validity. The guides were then pilot tested with five participants (two patients, two caregivers, and one health worker) at St. Paul’s Millennium Medical College, a non-study site, to assess clarity, cultural appropriateness, and length. Minor wording adjustments were made following the pilot test. The guides were designed to explore determinants both enablers and barriers across various levels, including individual, interpersonal, community, organizational, and policy dimensions. Separate interview guides were developed for each participant category: patients, caregivers, community leaders, health professionals, and policymakers. Each interview guide contained open-ended questions followed by probing prompts to elicit detailed narratives. Example questions included: “Can you tell me about your experience with palliative care services?” and “What factors have helped or hindered your ability to receive or provide palliative care?” (Supplemanatry Material_2).

The selection, recruitment, and data collection processes were led by a qualitative research expert in collaboration with the primary investigator. Senior researchers closely monitored the entire data collection procedure. We engaged study participants with the support of local community health workers who served as community liaisons. These health workers introduced the research team to potential participants, explained the purpose of the study, and facilitated initial contact. After establishing initial contact and conducting introductory discussions, we obtained their consent to proceed with formal research activities.

All interviews were conducted in a private, quiet location chosen by the participant, typically either at the health facility in a private room or at the participant’s home. Interviews lasted between 45 and 90 min, with an average duration of 60 min. No repeat interviews were conducted. We used an audio recorder and took field notes to capture all discussion points. Field notes included observations about participants’ non-verbal communication, the interview environment, and the researcher’s reflections. Participants were selected gradually based on preliminary analysis, ensuring maximum variation by considering factors such as age, sex, educational level, type and stage of cancer, urban versus rural settings, and the professional backgrounds of key informants. Data collection continued until thematic saturation was reached, meaning that subsequent interviews yielded no new codes or themes.

### Ensuring trustworthiness

To ensure the rigor and quality of this qualitative study, we applied the four criteria proposed by Lincoln and Guba: credibility, dependability, confirmability, and transferability.

To enhance credibility, we built rapport with participants through introductory conversations before formal interviews and allocated sufficient time (up to 90 min) for open dialogue. The primary investigator engaged with each study setting for two weeks prior to data collection. Member checking was conducted with ten participants, who reviewed a summary of preliminary findings to confirm accurate interpretation of their perspectives. Weekly peer debriefing sessions were held with co-authors not involved in data collection to review emerging findings and challenge interpretations. For dependability, we maintained an audit trail documenting all research decisions, including interview guide modifications, sampling choices, and analytical steps. Regular debriefing sessions with supervisors reviewed consistency between data and findings, and an external qualitative researcher reviewed 20% of transcripts and the coding framework to assess consistency. To ensure confirmability, all study documents were meticulously archived. Audio recordings were transcribed verbatim within 48 h, with transcripts checked against original recordings. A reflexivity journal documented the primary investigator’s assumptions and potential biases, which were discussed during team meetings to minimize their influence on findings. Transferability was promoted through thick description of study contexts, including detailed information about the two sites, participant characteristics, and the health system environment, enabling readers to assess applicability to their own settings.

### Data management and analysis

A framework analysis of qualitative data guided by the socio-ecological model was applied. Framework analysis was selected because it is particularly appropriate for health policy and systems research where the aim is to generate actionable recommendations. This approach involves five iterative stages: familiarization, identifying a thematic framework, indexing, charting, and mapping and interpretation. The SEM provided the a priori thematic framework, which was then refined through inductive coding of participant narratives to identify specific determinants within each level (Table 1).

**Table 1:** Framework analysis for determinants of comprehensive palliative care delivery among female cancer patients in Ethiopia

Theme(SEM Level)	Sub-themes ( Determinant)	Codes
Individual	1. Personal Health Literacy & Assets	Active information-seeking; Lack of awareness; Misconceptions about cancer; Fatalistic views; Belief in curses as cause
	2. Economic Resources & Financial Capacity	Catastrophic out-of-pocket costs; Indirect costs (transport, accommodation); Asset depletion; Treatment abandonment
	3. Psychological & Spiritual Assets	Personal resilience; Religious faith and holy water use; Loss of autonomy; Dependency on others; Trust in medical system
	4. Physical Health & Comorbidity	Advanced stage at diagnosis; Co-existing illnesses; Bedridden status
Interpersonal	1. Family Support & Caregiving Dynamics	Supportive spouse/children; Extended family support; Neglect and abandonment; Partner refusal of treatment
	2. Family Stigma & Discrimination	Cancer as punishment; Contagion beliefs; Social isolation within family; Non-disclosure
	3. Provider-Patient Communication & Relationship	Compassionate communication; Abrupt diagnosis disclosure; Disrespectful care; Lack of information; Physician-centric model
	4. Peer Support among Patients	Shared experiences; Emotional strength; Practical advice; Material support
Community	1. Social Capital & Community-Based Support Networks	Survivor-led support groups (Nigat); Edirs (traditional burial associations); Religious leaders; Community volunteers; Beza for Generation
	2. Traditional Structures & Healers	Traditional healers; Holy water (Tsebel); Herbal remedies; Bridging gaps in medical care
	3. Social Stigma & Community Rejection	Cancer as contagious; Isolation by neighbors; Fear of disclosure; Discrimination
	4. Fragility & Unsustainability of Support	Lack of resources; Poor coordination; Community fatigue; Episodic vs. organized support
Organizational	1. Access to Healthcare Services	Specialized cancer centers (TASH, HUCSH-CTC); CBHI scheme; Decentralization of care; Geographic disparities
	2. Organizational Culture & Prioritization of Palliative Care	Systemic undervaluation of PC; Threat of unit closure; Low priority from administrators; Lack of institutional support
	3. Resource Allocation & Material Scarcity	Opioid shortages (morphine, tramadol); Lack of trained staff; No multidisciplinary teams; Broken referral systems; Long waiting times
Policy	4. Service Delivery Models	Facility-based care; Home-based palliative care (absence of); Health Extension Workers (underutilized); Family health teams
	1. Governance & Political Will	Existence of national PC guideline; Inclusion in government health agenda; Family Medicine ownership; Advocacy tool
	2. Regulatory Frameworks for Opioids	Restrictive opioid regulations; No local morphine production; Interrupted production initiative; Fear of addiction among prescribers
	3. Health Professional Education Policies	Absence of PC in curriculum; No on-job training; Shortage of trained professionals; Lack of expertise
	4. Policy Gaps & Implementation Challenges	Outdated guideline (7–8 years old); Theoretical vs. practical; No level-specific guidance; No rural implementation strategy

Data management and analysis were conducted concurrently with data collection, following the five stages of framework analysis using ATLAS. ti V.09.

Stage 1: Familiarization: We transcribed the audio recordings verbatim in the original language (Amharic or Sidama) within 48 h of each interview. Each transcript was read multiple times by two researchers independently to gain immersion in the data. During this stage, researchers wrote memos about initial impressions and potential themes.

Stage 2: Identifying: a Thematic Framework. An initial coding framework was developed based on the five levels of the Socio-Ecological Model (individual, interpersonal, community, organizational, policy). Within each level, we inductively identified sub-themes that corresponded to key determinants of health, such as personal health literacy, financial resources, social support networks, community social capital, organizational resource allocation, and governance/regulations. This a priori framework was then supplemented with inductive codes that emerged from the data. The updated codebook was reviewed and refined by the research team after coding the first ten transcripts.

Stage 3: Indexing: The transcribed documents were uploaded to ATLAS. ti V.09 software for systematic coding. Two coders (SS and NS) independently coded a subset of transcripts (30%, *n* = 28) to assess inter-coder reliability. The initial agreement rate was 82%; disagreements were resolved through discussion with KS, and the codebook was clarified accordingly. The remaining transcripts were coded by the primary coder with regular checks by the second coder.

Stage 4: Charting: Coded data were then charted into framework matrices. Separate matrices were created for each SEM level, with rows representing participants and columns representing codes. Data were summarized from the original transcripts into these matrices, preserving the participants’ own language where possible while reducing volume for analysis.

Stage 5: Mapping and Interpretation: The final stage involved mapping the interpreted data to address the research questions. Patterns, convergences, and divergences across participant groups (patients, caregivers, health workers, policymakers) and across sites (Addis Ababa vs. Sidama) were systematically compared. Themes were developed by clustering related codes, and relationships between themes were examined to build a coherent narrative about the determinants of palliative care. Representative quotations were selected to illustrate each theme, with attention to including voices from different participant categories and both study sites.

Throughout the analysis, reflexivity was maintained through regular daily team debriefings. The research team met daily during the analysis phase to discuss emerging findings, challenge assumptions, and ensure that interpretations remained grounded in the data. Coding was conducted by two coders independently (SS and NS) on a subset of transcripts to enhance consistency, with differences resolved through discussion with KS. Themes have been developed by clustering related codes, and the relationships between themes were examined to build a coherent narrative about determinants. Data from different sources, such as patients, caregivers, community leaders, health workers, and key informants have been compared to identify convergences and divergences in perspectives. Negative or deviant cases, where participant accounts did not fit the dominant pattern, were actively sought and analyzed to refine and qualify the findings.

### Findings

#### Study participants’ profile

A total of 93 participants took part in this study, comprising 37 women with breast or cervical cancer, 20 caregivers, and 36 key informants. Among the 37 patients, 20 had breast cancer and 17 had cervical cancer. Their ages ranged from 27 to 70 years. Twenty patients were married, while the remainder were widowed, divorced, or single. Regarding education, 15 had no formal education, 10 attended elementary school, 7 completed secondary school, and 5 held diplomas or degrees. The majority were unemployed or housewives. Seventeen patients had lived with their cancer diagnosis for three or more years, while 10 had been diagnosed within the preceding two years. Among the 20 caregivers, 12 were female. Their ages ranged from 22 to 55 years. Educational levels varied: four had no formal education, six completed elementary or secondary school, seven held diplomas, two had degrees, and one held a master’s degree. Caregivers included sons, daughters, spouses, siblings, friends, and volunteers. The 36 key informants comprised religious leaders, edir leaders, social workers, nurses, physicians, program coordinators, health extension workers, and policy experts from federal, regional, and local levels. Their ages ranged from 25 to 72 years; 23 were male and 13 female. Most held degrees or master’s degrees and were government employees with professional experience in palliative care or cancer services (Table 2).

**Table 2:** Socio-demographic profile of study participants (*N* = 93)

Characteristic	Patients (*n* = 37)	Caregivers (*n* = 20)	Key Informants (*n* = 36)
Age (years)	27–70	22–55	25–72
Sex			
Female	37	12	13
Male	0	8	23
Marital status			
Married	20	10	22
Widowed	7	4	2
Divorced	6	2	3
Single	4	4	9
Education			
No formal education	15	4	0
Elementary (Grade 1–8)	10	6	1
Secondary (Grade 9–12)	7	4	2
Diploma	3	7	8
Degree	2	2	15
Master’s or above	0	1	10
Occupation			
Unemployed/housewife	30	5	0
Government employee	2	2	28
Private worker	5	8	5
Religious/community leader	0	0	3
Cancer type (patients only)			
Breast cancer	20	-	-
Cervical cancer	17	-	-
Time since diagnosis (patients)			
< 1 year	10	-	-
1–2 years	10	-	-
3–5 years	12	-	-
> 5 years	5	-	-
Caregiver relationship			
Spouse	-	3	-
Child (son/daughter)	-	7	-
Sibling	-	4	-
Volunteer/friend	-	6	-
Key informant role			
Religious/Edir leader	-	-	9
Health professional (nurse, MD, specialist)	-	-	14
Program coordinator/administrator	-	-	8
Health extension worker/social worker	-	-	5

### Framework analysis using socio-ecological model

The study explored the multi-level determinants influencing the implementation of comprehensive palliative care for women with breast and cervical cancer in Ethiopia. The findings are organized and presented using the Socioecological Model framework, detailing determinants across five interconnected levels (Table 3).

**Table 3:** Multi-level determinants for comprehensive palliative care delivery. SEM: Socioecological Model

SEM Level	Enabling Determinants	Hindering Determinants
Individual	Personal health literacy; psychological resilience; religious faith; trust in medical care	Lack of awareness, catastrophic out-of-pocket costs, misconceptions (cancer as curse/contagious), dependency on others, comorbidity
Interpersonal	Compassionate provider communication; peer support; supportive families; supportive partners	Poor partner support (neglect/abandonment), family stigma, poor provider communication, fear of opioid addiction among providers
Community	Survivor support groups; edirs (traditional insurance); religious leaders; community volunteers; traditional healers	Social stigma (contagion belief), resource deficits, poor coordination, community fatigue
Organizational	CBHI scheme; specialized cancer centers; Hospice Ethiopia; limited training opportunities	Systemic undervaluation of palliative care; medicine shortages (opioids), lack of trained staff, no multidisciplinary teams, long waiting times, poor referral systems; no home-based care
Policy	National palliative care guideline exists; government health agenda; Family Medicine ownership	Outdated policy, no strategic plan contextualizing local resources, restrictive opioid regulations, no local morphine production, no palliative care curriculum

1. Individual level: Personal health literacy, economic resources, and psychological assets.

At the individual level, the capacity to engage with palliative care was shaped by the patient’s personal assets, beliefs, and socioeconomic circumstances. Key determinants included personal health literacy, financial resources, and psychological/spiritual assets.

### Personal health literacy

As the study showed, patient agency, largely driven by health literacy, was a significant enabling determinant. Patients with a background in health professions or those who were educated and actively sought health information reported a better understanding of their condition and medical interventions. Both breast and cervical cancer patients described how this knowledge helped them communicate more clearly with healthcare providers, follow treatment plans, and cooperate with caregivers at home. As a breast cancer patient from TASH who was a health professional explained:

“Thanks to God, there is nothing that is missing for me. As I am a health professional, I have no problem in communication and getting services it helped me indirectly”.

As the finding explained, patients in Addis Ababa generally demonstrated higher health literacy and more active information-seeking behaviors compared to patients from Sidama. A key informant from Hospice Ethiopia noted:

“Patients from Addis Ababa usually ask questions and want to understand their treatment, but those coming from far rural areas often do not know what cancer means. We have to start from the very beginning, explaining what the disease is and why they need treatment.”

Conversely, as articulated by participants, a lack of awareness and widespread misconceptions about cancer were major hindering determinants. Many patients and caregivers viewed palliative care as a curative service, leading to unrealistic expectations when rapid progress was not observed. Misconceptions often framed cancer as a communicable disease, a curse, or divine punishment, which eroded trust in palliative care. Many patients internalized a fatalistic view of cancer, believing it was a death sentence, which led to emotional instability and initial refusal of medical interventions and care. As a cervical cancer patient from Hawassa explained:

“I believe that cancer is a curse and I don’t think the service I get from Hospital will help me”.

As the finding showed, in Sidama, misconceptions about cancer being a curse or divine punishment were more dominant compared to Addis Ababa. A religious leader from Sidama explained:

“In our community, many people believe that cancer comes from a curse or as punishment for wrongdoing. They often take patients to traditional healers first and only come to the hospital when the condition is very advanced.”

### Economic resources and financial capacity

As stated by participants, financial constraints were a primary hindering determinant at the individual level which hindered patients and caregivers from getting the service they wanted. The costs of investigations, medications, food, and transportation were described as catastrophic. Many patients lost their livelihoods due to illness, and their families’ incomes were compromised as caregivers took time off work to care for the patient. Patients and caregivers explained that due to the financial hardship, they sold assets, including homes, to get care service, and were burdened with worry for their families’ futures. The financial difficulty caused many patients to skip medications or abandon treatment and care altogether. As a caregiver from Hawassa explained:

“We are all quite poor. For instance, my daughter come in to hospital with pain, but we are more concerned about food and her children’s schooling. Therefore, if we cannot get support for this, we cannot give proper care for her and ourselves”

As the finding showed, while both patients from Addis Ababa and Sidama faced huge out-of-pocket expenses, patients from Sidama incurred additional costs for long-distance travel, accommodation in Addis Ababa or Hawassa for extended periods, and lost income from time away from farming or daily labor. A breast cancer patient from a rural area who had become bedridden and could not afford to continue treatment stated:

“I believe if I had enough money, I could eat enough food, get medication, and perhaps be recovered”

### Psychological and spiritual assets

As mentioned by participants, personal resilience and religious faith were enabling determinants. Many patients drew upon inner strength, often supported by religious faith, to accept their diagnosis and cope with the associated challenges. Caregivers mentioned that those patients who accepted their case and were emotionally strong sought environments and relationships that provided support and practical coping strategies. Furthermore, trust in medical care and healthcare providers was also mentioned as a motivator.

However, as articulated, loss of autonomy due to total dependency on others for care and decision-making was a hindering determinant. This often prevented patients from accessing the specific support they felt was important to their well-being. As cervical cancer patient from siddama explained:

“My family rushed me to chemo but silenced my soul; the healing I begged for was in my faith, not just in my veins”

As described, this loss of autonomy was particularly pronounced among patients of Sidama-origin who traveled to Addis Ababa for treatment at Tikur Anbessa Specialized Hospital. These patients became completely dependent on caregivers or fellow patients for navigating the unfamiliar urban environment including arranging transport, finding accommodation, and managing daily logistics which contrasted with their greater autonomy and familiarity in their home communities. A breast cancer patient from a rural area whose husband had left her explained:

“I had no one to accompany me to the hospital and could not continue follow-up since I don’t have money. As you see I am bedridden at home…”

2. Interpersonal level: Social support, family dynamics, and provider-patient relationships.

At the interpersonal level, the immediate social environment family, partners, and healthcare providers represented a critical level where determinants operate. Key determinants included social support networks, family caregiving dynamics, and the quality of provider-patient relationships.

### Social support networks

As described by patients and caregivers, supportive families and compassionate providers were enabling determinants. In both Addis Ababa and Sidama, supportive families were described as enablers. However, the nature of family support differed. In Addis Ababa, patients more often reported support from spouses and adult children who lived nearby and could accompany them to appointments. In Sidama, patients often relied on extended family networks including siblings, cousins, and neighbors, as immediate family members were sometimes engaged in farming or other income-generating activities far from the health facility. As a caregiver from Sidama whose wife had breast cancer explained:

“…he could not be with her throughout all medical processes, as he had to go to the farm to cover expenses”.

Conversely, as stated, poor partner support, including neglect and abandonment, was a devastating hindering determinant that created additional trauma for many breast and cervical cancer patients. Patients were left to manage not only their illness but also household responsibilities and childcare, exacerbating their stress and isolation. As a breast cancer patient from TASH stated:

“My partner’s neglect hurt almost as much as the cancer. I was left to deal with everything by myself my sickness, the house, our kids. It made me feel so alone”.

As the finding showed, in Sidama, stigma was reported as more intense and more openly expressed compared to Addis Ababa. A cervical cancer patient from a rural area described:

“My husband and children were not well aware about cancer and used to stigmatize me thinking that the cancer was caused by sexual activities like HIV/AIDS and doubted that I might have been with another partner”.

### Provider-patient relationship dynamics

As articulated, compassionate communication from healthcare providers created a welcoming environment that encouraged patients to adhere to advice and utilize available services. As health care providers explained, when patients feel listened to and cared for, they trust providers more.

“The trust they have on HCP is what helps patients to follow through with their treatment and stay involved in their own care” (HCP, Hawassa).

However, as mentioned, poor communication from healthcare providers was a major hindering determinant. Patients reported a lack of adequate information about their diagnosis, disease progression, treatment, and care options. Abrupt disclosure of diagnosis, along with disrespectful care by hospital staff, enhanced frustration and disengagement. A cervical cancer patient from a rural Siddama area explained how a doctor’s approach led to despair:

“The doctor said that this treatment is also given at Tikur Anbesa… I was very shocked at that moment”

3. Community level: Social capital, community networks, and traditional structures.

At the community level determinants included social capital, community-based support networks, and the role of traditional and religious structures.

### Social capital and community-based support networks

As the study found, informal social support mechanisms, though fragmented and hampered by stigma and resource limitations, served as enabling determinants. Support groups, particularly those led by cancer survivors, provided emotional and practical support. As community members explained, they mobilized the community for financial and material aid to cover patients’ medical expenses, transportation, and food. As breast cancer patient and survivor group member from AA articulated:

“The Nigat group, made up of survivors, is so important because patients get strength from talking to others who have been through the same case. Getting advice from a peer who truly understands is the best way to cope with the loneliness and hopelessness”.

As articulated, in Addis Ababa, formal survivor-led support groups like Nigat operated with regular meetings, trained peer counselors, and some external funding. In Sidama, community support was more informal and episodic, relying heavily on traditional structures such as edirs and religious institutions, as well as local organizations like Beza for Generation.

The evolving role of Edirs (traditional community insurance/burial associations) was mentioned as a notable enabling determinant. In Sidama, Edirs had shifted their focus from solely supporting bereaved families to providing assistance to living members, helping to cover treatment costs and transportation. As Edir leader from sidamma explained:

“Edirs don’t wait for them until the loss of their life as before. For example, let me tell you about our work in the area of cancer. We tried to identify four mothers and send them to undergo the test at Yirgalem hospital… So, we tried to cover their transportation and medical costs when they started the treatment”.

However, as described, a key informant from Beza for Generation explained the fragility of this support:

“We used to support patients by mobilizing community members… However, this support stopped about five years ago due to lack of funds, and currently it is done only based on individual effort, not organizationally”.

As stated, religious leaders and community volunteers played a crucial role in offering spiritual support to patients, educating the community to reduce stigma, and providing a link between patients and formal health services. As a religious leader from Yirgalem described:

“I mobilize people around me to contribute money for patients. Patients get food items, clothes, and sanitary materials. I also work with healthcare providers and link patients when I find they need medical support”.

### Social stigma

In this study, as described, social stigma, rooted in the belief that cancer is a curse and contagious, led to profound social isolation of patients and their families. Patients reported being discriminated against by neighbors and friends, which exacerbated their stress and hopelessness and limited their social participation. As a cervical cancer patient from TASH explained:

“People acted like cancer was catching. Old friends pulled away, neighbors whispered it left us more alone than the disease itself…the worst part wasn’t the illness; it was how our community treated us. The fear and stigma pushed everyone away when we needed them most”.

As described, stigma was very visible in Sidama. A religious leader from Sidama explained: “Patients do not disclose their status due to fear of what others might talk about their condition. This prevents Edirs from knowing who needs support”.

### Difference in individual, interpersonal, and community level determinants of breast, and cervical cancer patients

The study showed that, at the individual level cervical cancer patients experienced more pronounced burden of stigma rooted in the association of cervical cancer diagnosis with sexually transmitted infections. Several cervical cancer patients both from Addis Ababa and Sidama reported that family members assume infidelity or risky sexual activity as the cause. This led to heightened shame and social isolation. As cervical cancer patients from a rural area described:

“My husband and children were not well aware about cancer and used to stigmatize me thinking that the cancer was caused by unwarranted sexual activities and suspected that I might have been with another man”,

However, this association was not articulated by breast cancer patients, whose stigma was more commonly linked to fears of contagion or visible disfigurement following breast removal.

At the interpersonal level, partner abandonment was reported by both groups. Among cervical cancer patients, abandonment was frequently tied to perceptions of sexual impropriety that leads to husband’s withdrawal of support. In contrast, breast cancer patients explained partner’s neglect linked to physical changes and loss of femininity following breast surgery and financial burden of prolonged treatment. Breast cancer patients from Addis Ababa expressed that;

“My partner’s neglect hurt almost as much as the cancer. I was left alone to deal with everything by myself, my sickness, the house chores, our kids. It made me feel lonely and depressed”.

At the community level, stigma manifested differently for the two cancers. For cervical cancer patients, community-level stigma was openly expressed as rooted in the belief that the disease was caused by promiscuity. This led to community rejection, gossip, and social exclusion, with neighbors and community members distance themselves from patients and their families. Religious leader from Sidama explained that;

“Patients do not disclose their status due to fear of what others might talk about their condition…”.

For breast cancer patients, community stigma was associated with fears of contagion, and consequent avoidance of physical contact or isolating patients due to misconceptions about cancer being a communicable disease. A breast cancer patient from Addis Ababa described:

“People acted like cancer was catching. Old friends pulled away, neighbors whispered it left us more alone than the disease itself.”

Breast cancer patients generally described their disease and treatment options, partly due to more visible public awareness campaigns and survivor advocacy. However, cervical cancer patients frequently expressed misconceptions about causation and lacked awareness of the link between HPV and cervical cancer. A cervical cancer patient from Hawassa explained:

“I believe that cancer is a curse and I don’t think the service I get from the hospital will help me.”

Despite these variations in stigma, partner abandonment, and awareness between cervical and breast cancer patients, both groups shared common challenges, including catastrophic out-of-pocket costs, long-distance travel for care, and the critical role of supportive families in enabling treatment adherence and palliative care access. As a caregiver from Sidama noted:

“ My daughter came to the hospital with pain, but we the family are more concerned about food and her children’s schooling. Therefore, if we can not get support for this, we cannot give proper care for her and ourselves.”

4. Organizational level: Access to healthcare, resource allocation, and organizational culture.

At the organizational level, the determinants were primarily structural, including access to healthcare services, organizational resource allocation, and the prevailing organizational culture regarding palliative care.

### Access to specialized services and community based health insurance (CBHI)

As described, the establishment of specialized cancer centers in Addis Ababa and Hawassa was an enabling determinant, decentralizing care and reducing travel burdens for many. A nurse from Hawassa stated:

“The establishment of the cancer center itself gives relief because it includes a designated unit for palliative care which the care is too expensive at private facilities”.

As articulated by a health worker from Hawassa;

“ The presence of a cancer center in Hawassa was enabler for patients from Sidama as before this center opened, patients from Sidama had to go all the way to Addis Ababa. Many could not afford the transport or find a place to stay there. They simply gave up. Now, at least they can come here. It is still far for some, but it is much closer than Addis”.

As described the Community-Based Health Insurance (CBHI) scheme, despite its limitations, reduced out-of-pocket costs for some patients accessing public facilities. Furthermore the dedicated work of organizations like Hospice Ethiopia, which provided home-based and facility-based care, was also a crucial enabling determinant. However, as noted, Hospice Ethiopia operated primarily in Addis Ababa, with limited outreach to Sidama, creating a geographic disparity in access to organized palliative care services. As a Hospice Ethiopia staff member explained:

“As Hospice Ethiopia, we try to solve patients’ social and economic issues. We have food support and a comfort fund for financial support. We provide food support for 40 people every 3 months, and we provide monetary support for 45 people every month. We also have a daycare program for ambulatory patients every two weeks on Thursday”.

### Organizational culture and resource scarcity

As stated, the main organizational barrier was the systemic undervaluing of palliative care within the health system. Palliative care units were often the lowest priority for hospital administrators, leading to a lack of institutional support, frustrated staff, and poor service quality. As a healthcare worker from Hawassa explained:

“…they think of closing this palliative care unit, considering as it is not necessary… So nobody is giving attention to it”.

As the finding showed, in Addis Ababa, despite having more resources and specialized staff, the palliative care unit faced threats of closure due to perceived low priority. In Sidama, the palliative care unit at Hawassa Cancer Center was understaffed and underequipped. An oncologist in TASH acknowledged:

“The integration of palliative care into the health system is weak… Lack of resources, and low priority is the biggest challenge.”

This undervaluation was compounded by severe resource scarcity. Health facilities faced severe deficits in trained human power, funding, and physical space. Shortages of essential medicines, particularly opioids like morphine for pain management, were repeatedly emphasized. As family medicine physician from TASH stated:

“Palliative care is resource-intensive; it requires a lot of resources, so it is difficult to work with the existing situation as well, because there is no medication, there is no professional, there is no material, and a conducive environment”.

As described, the medication shortage was very serious in Sidama. A nurse from Hawassa Cancer Center explained: “In Addis, patients can sometimes buy medications from private pharmacies near the hospital. Here in Hawassa, even the private pharmacies often do not have what patients need. We have to send patients to Addis Ababa just to buy medications, which adds to their travel costs and suffering”.

The lack of home-based palliative care was a critical hindering determinant, particularly for patients in Sidama, where long distances to health facilities made regular hospital visits impossible for bedridden patients. As a health extension worker from Sidama explained:

“We have patients who cannot leave their homes because they are in too much pain. The hospital is two hours away by car. I wish I could visit them at home, and provide medication”.

5. Policy level: Governance, regulations, and health system financing.

At the policy level, the overarching determinants included governance and political will, regulatory frameworks for opioids, and health professional education policies.

### Policy foundation

As described, the existence of a national palliative care guideline and its inclusion in the government’s health agenda were enabling determinants, providing a legitimate basis for palliative care initiatives and advocacy. Efforts to assign ownership of palliative care to the Family Medicine department were also seen as a positive step towards creating a clear focal point for service development and training. As a policy expert stated:

“The guideline is not perfect but without it there would be no palliative care at all, so it is a tool to push the decision makers to provide value for palliative care…”.

### Regulatory and curriculum gaps

As articulated, the national palliative care guideline was widely reported to be outdated, overly theoretical, and lacking practical, context-specific operational guidance for different levels of the health system. As a key informant from Ministry of Health explained:

“I think it is the first version, and I would say it is old. Unfortunately, we didn’t revise it, I think it has been 7–8 years since it was endorsed….The other thing is that it doesn’t designate what services shall be given at each level of hospitals… So it needs revision”.

As articulated, the most frequently mentioned policy barrier was the lack of local morphine production and restrictive regulation of opioids. Stringent controls made it difficult for facilities to stock and for physicians to prescribe essential pain medications like morphine, rendering effective pain management nearly impossible. Hospice Ethiopia, for instance, was prohibited from handling opioids, hugely limiting its effectiveness. As palliative care provider from Addis Ababa explained:

“After assessing a patient’s pain, we cannot provide medication. Current restrictions prohibit opioids, and physicians now avoid tramadol and morphine. This has led to market unavailability. A local morphine production initiative was interrupted. I urge for immediate attention to restart production and ensure these essential pain medications are accessible”.

As described, the opioid shortage was felt more acutely in Sidama, where alternative pain management options were even more limited. A nurse from Hawassa Cancer Center explained: “In Addis, at least some private pharmacies might have tramadol or other strong painkillers. Here, often there is nothing. Patients with severe pain are told to go to Addis to buy medication, which most cannot afford. So they suffer in silence at home”.

The absence of on-the-job training and a dedicated palliative care curriculum in the education of health professionals resulted in a shortage of trained manpower. Most health graduates entered the workforce with little to no formal training in palliative care, resulting in a lack of expertise and a misunderstanding of its scope among practicing healthcare providers. As a key informant from Hospice Ethiopia explained:

“The other gap is that palliative care is not included in the curriculum. Again, we don’t have nurses and physicians who have special training in palliative care. If you count professionals like me who took a diploma training, you may not count more than 2–3 people”.

As a clinical nurse from Yirgalem Health Center also noted “I have never received training on palliative care. I don’t know the national palliative care guideline…”.

## Discussion

Guided by the Socio-Ecological Model, this study provides a multi-level analysis of the determinants of comprehensive palliative care for women with breast and cervical cancer in Ethiopia. The findings reveal a complex, interacting system of determinants across five levels. The overarching narrative is that while enabling determinants exist at all levels, they appear to be systematically undermined by hindering determinants, particularly at the organizational and policy levels. Furthermore, the study illuminates how determinants at one level seemed to shape conditions at another, creating a cascade of advantage or disadvantage.

At the individual level, a patient’s personal health literacy (an enabling determinant) was perceived to be profoundly shaped by the policy level (e.g., absence of a palliative care curriculum in schools) and the organizational level (e.g., lack of patient education materials or time for providers to counsel). As the study showed, a health professional patient had high health literacy and navigated care well, while a non-educated patient from a rural area lacked basic awareness and faced catastrophic financial consequences. This financial catastrophe at the individual level was exacerbated by organizational failures (e.g., medications not available in public hospitals, forcing purchase from expensive private pharmacies) and policy failures (e.g., CBHI not covering all essential drugs, restrictive opioid regulations limiting availability). This finding is consistent with studies that identified awareness, norms and poverty as factors affecting palliative care service [19, 20, 21].

The interpersonal level shows a similar pattern of interaction. Stigma from family, a powerful hindering determinant, was reported to be more intense in Sidama. This family-level stigma appeared to be a manifestation of broader community-level social norms and misconceptions about cancer as a curse or contagious disease. Community-level stigma appeared to reinforce the interpersonal dynamic, leading to patient isolation and non-disclosure, which in turn seemed to prevent patients from accessing organizational-level services and community-level support from edirs. This finding aligns with research from other Sub-Saharan African countries where female cancers like cervical cancer are particularly stigmatized due to their association with sexually transmitted infections [22, 23].

The community level reveals how social capital can function as both an asset and a liability. The traditional structure of edirs is an enabling determinant rooted in Ethiopian culture. However, the study showed that edirs in Sidama are evolving to support living cancer patients, a positive adaptation. Yet, their impact appeared to be constrained by a lack of financial resources and by stigma that prevents patient disclosure. Community owned organization in Sidama provided crucial support, but its closed due to lack of funds was interpreted as reflecting a policy failure regarding sustainable financing, leaving a vacuum. This finding suggests how the absence of supportive policy (e.g., a national funding mechanism for community-based palliative care) may undermine effective community and organizational-level determinants, a finding consistent with studies on sustainability challenges facing community-based organizations in resource-limited settings [24, 25].

Cervical cancer patients experienced stigma rooted in association with sexually transmitted infections, leading to shame and social exclusion, while breast cancer patients’ stigma was linked to fears of contagion or visible disfigurement. Partner abandonment was reported by both groups, but driven by different factors like perceptions of sexual impropriety for cervical cancer and physical changes and loss of femininity for breast cancer. Differences in awareness was also the case where breast cancer patients demonstrated relatively better awareness, while cervical cancer patients expressed misconceptions about causation. Studies from other Sub-Saharan African countries has documented differences on awareness about cervical and breast cancer [22, 23]. This highlights the need for cancer-specific interventions. Cervical cancer programs should prioritize community sensitization on STI misconceptions and family counseling, while breast cancer interventions should address contagion fears, provide psychosocial support, and leverage survivor networks.

The organizational level emerged as the critical nexus where higher-level policies translate into service delivery. The presence of a specialized cancer center in Hawassa was a major enabling determinant. However, its impact appeared to be limited by the policy-level failure to integrate palliative care into the curriculum and to liberalize opioid regulations. The shortage of trained staff and essential medicines at the organizational level perceived as consequences of policy-level decisions. Furthermore, the systemic undervaluation of palliative care within hospital administration (an organizational culture determinant) may be linked to a policy-level lack of mandated standards, dedicated budgets, and monitoring indicators. This finding is comparable with studies conducted in Africa that highlight the critical undervaluing of palliative care within health systems [15, 20, 26].

Finally, the policy level emerges as the overarching determinant. The national guideline and government agenda are enablers, but their impact appeared to be negated by outdated content, restrictive opioid laws, and the absence of palliative care from educational curricula. These policy failures seemed to create a cascade of barriers down the level from poorly trained providers (organizational), to unaffordable and unavailable medications (organizational), to community programs lacking sustainable funding (community), to families who lack knowledge and stigmatize patients (interpersonal), and finally to individuals who face catastrophic costs without adequate pain relief. This aligns with research conducted in Ethiopia [14, 15, 25] and abroad [19, 27] that identifies policy gaps as fundamental barriers.

The urban-rural disparities observed throughout the findings greater stigma, lower health literacy, more catastrophic indirect costs, weaker organizational capacity, and less access to specialized services in Sidama appeared to be a function of how these multi-level determinants interact and concentrate in different geographic contexts. This finding is consistent with studies from other Africa countries showing that urban residence is associated with greater health literacy and earlier care-seeking for cancer [28, 29]. The study implies that without targeted interventions that address these interacting determinants across all levels, comprehensive and equitable palliative care cannot be achieved.

### Strengths and limitations of the study

The application of the Socio-ecological model provided a framework to organize the complex, multi-level determinants influencing comprehensive palliative care delivery. The large sample size (*n* = 93) provided sufficient data to capture full variation of experiences across different participant groups, allowing for a more nuanced understanding of the determinants. The inclusion of diverse stakeholder groups (patients, caregivers, providers, community leaders, and policymakers) and the selection of urban, semi-urban, and rural sites enhanced the richness and transferability of the findings within the Ethiopian context. Researcher positionality was transparently managed through reflexivity practices including journaling, audit trails, and team debriefings. This enhanced the credibility and rigor of the findings.

However, the study also has limitations. Findings are based on self-reported data, which may be subject to recall and social desirability biases. While saturation was achieved, the multi-site, multi-stakeholder nature of the sample means saturation should be interpreted cautiously. Additionally, interviews were conducted in Amharic and Sidama and transcribed in the original languages before being translated into English for analysis and reporting. This translation process may have resulted in subtle loss of cultural nuances, or emotional depth embedded in participants’ original expressions, despite efforts to ensure accuracy through careful translation and review by bilingual researchers. Finally, Pastoralist communities were not included in the sample, therefore future study should explore palliative care determinants among pastoralist communities.

### Conclusion, and recommendations

This study provides multi-level analysis of determinants shaping comprehensive palliative care for women with breast and cervical cancer in Ethiopia. The findings reveal that while enabling determinants exist across all five socio-ecological levels, they are systematically outweighed by deeply rooted organizational and policy barriers.

To achieve equitable, comprehensive palliative care, synergistic actions are needed across all levels. Policy reforms must prioritize guideline revision with explicit rural implementation strategies, liberalization of opioid regulations, and mandatory integration of palliative care into health professional curricula. Organizational actions should include dedicated budgets, multidisciplinary teams, home-community-based care models, and structured training for Health Extension Workers. Community-level interventions must formalize and strengthen existing support mechanisms through sustainable funding models for edirs and religious institutions, alongside stigma reduction training.

The broader implication of this study is that without addressing the interconnected barriers across all levels, efforts to improve palliative care will remain fragmented and ineffective. The findings call for a paradigm shift from fragmented, facility-based, and urban-centric approaches to integrated, community-engaged, and decentralized models that prioritize the needs of the most vulnerable populations, particularly rural women.

## Supplementary Information

Supplementary Material 1.

Supplementary Material 2.

## Data Availability

All relevant data are within the manuscript, and the complete lists of the studies extracted and analyzed for this study will be available upon the reasonable request of first or corresponding author.

## Abbreviations

Ca: Cancer
CBHI: Community-Based Health Insurance
CxCa: Cervical cancer
Gov't employee: Government employee
HBC: Home-based care
HCP: Health care provider
HEWs: Health Extension Workers
HIV/AIDS: Human Immunodeficiency Virus / Acquired Immunodeficiency Syndrome
HUCSH: CTC-Hawassa University Comprehensive Specialized Hospital Cancer Treatment Center
IDI: In-depth interview
KII: Key informant interview
LMICs: Low-and middle-income countries
PC: Palliative Care
SEM: Socio-ecological model
TASH: Tikur Anbessa Specialized Hospital
UHC: Universal Health Coverage
WHO: World Health Organization
