# Supplementary material for: Multi-level determinants of comprehensive palliative care delivery for breast and cervical cancer patients in Ethiopia: using the socio-ecological model as an analytical framework
Source: BMC Palliat Care. 2026 Aug 25;25:257. doi: 10.1186/s12904-026-02300-z (PMC13504818; doi:10.1186/s12904-026-02300-z)
Supplement: Supplementary file 2 [file 12904_2026_2300_MOESM2_ESM.docx]

**Intervie guide**

**Objective**

- To explore the palliative care needs of female cancer patients, and their caregivers in Ethiopia
- To explore enablers, and challenges to improve the continuum of care through a comprehensive palliative care model for female cancer patients in Ethiopia

1. **For Patients**
A. **Socio-demographic and Clinical Characteristics**
1. **Can you tell me about yourself?**

**Probe:** age, educational level, marital status, job, source of income, family size, and living conditions?

2. **Can you tell me about your health status**

**Probe:** General health status, age at diagnosis of cancer?

- What type of cancer have you been diagnosed with?
- How long have you been living with this diagnosis?
- What stage is your cancer currently at?
- Are you currently receiving treatment?
- If so, what type?

3. **Understanding of Palliative Care and Care Experience**

- **Can you describe what palliative care is in your own words? Importance?**
- **Have you received palliative care services? Expectation and experience?**
- **Please tell me about the care you get?**

**Probe:** Types of care, frequency of care, source of care, when did you join?

- What aspects of the palliative care you received did you find most helpful or supportive and why? (physical, psychosocial, spiritual, economic, end of life)
- Specific experiences that made a positive impact on your quality of life?

B. **Palliative care need/experience**
4. **How have your symptoms been managed during your palliative care?**

**Probe:** Have you felt that your physical, Psychosocial, spiritual, and economical needs have been addressed?

a. Can you provide examples?
- Are there any specific treatments or interventions that have helped alleviate your symptoms? Is the medication easily available
- **What types of Psychosocial support do you feel you need most right now?**

**Probe:** How has your diagnosis impacted your relationships with family and friends?

- Are there any social services or community resources that you have found helpful?
- Do you have any spiritual or religious beliefs that are important to you during this time? Benefits?

5. **What are your thoughts or feelings about end-of-life care in general? What aspects of end-of-life care are most important to you (e.g., pain management, emotional support, spiritual care)?**

**Probe:** Are there specific settings (home, hospice, hospital) where you would prefer to receive end-of-life care? Why?

i. How comfortable do you feel discussing end-of-life care preferences with healthcare providers and family members?
ii. What types of support did you find most helpful during your bereavement process? Important/helpful experience

C. **Communication and Ethical Considerations**
6. **How would you describe your communication with your healthcare team regarding your palliative care needs?**

**Probe:** How did you get informed about your diagnosis at the health facility?

- How did you tell to your family/husband?
- Are there any barriers you face when discussing your needs or concerns with your providers? Did you feel well-informed about your palliative care options? Why or why not?
  i. How to get better communicated?
  ii. Information needed to make informed decisions about your care?
- How do you feel about the decision-making process regarding your treatment? Were you involved in discussions about your care options? In your opinion, what are the most important ethical or moral issues in palliative care? How can families and communities better support female cancer patients in accessing palliative care?
- Were there any moments when you felt your need were not respected? Can you share that experience?
- Have you ever felt dilemmas raised in your care? If so, can you describe one?
- Do you feel comfortable discussing sensitive topics (e.g., end-of-life issues) with your healthcare team? Why or why not?

D. **Preference on Palliative care delivery approaches/models**
7. **How would you define home-based care, community-based care, and facility-based care?**
8. **How did healthcare providers, family, or community members support you during your palliative care journey**? Any specific beneficial program?
9. **Which type of care setting do you prefer (home-based, community-based, or facility-based or else)? Why?**
10. **What do you see as the benefits of receiving care at home? A good example, challenges?**
11. **What do you think are the advantages of community-based care?**

**Probe:** Have you accessed any community resources or services? Example and barrier?

12. **What do you perceive as the benefits of receiving care in a facility**?

**Probe:** Can you share any concerns or drawbacks you associate with facility-based care?

i. Under what circumstances would you consider facility-based care to be necessary or preferable?

13. **What changes would you like to see in the way care is provided across home-based, community-based, and facility-based settings?**
14. **What role do your family and friends play in your decision-making regarding care options?** **Probe:** How important is the availability of healthcare professionals when considering your preferred care setting?
    a. Any specific services or amenities that would influence your choice of care setting?
15. **Is there anything else you would like to share regarding your needs or experiences related to palliative care?**

**Probe:** How can caregivers, volunteers, healthcare providers better support patients and families during this time?

E. **Enablers/opportunities to access palliative care**
16. **What opportunities exist to enhance palliative care services?**

**Probe:** (e.g., trained caregivers, community leaders, community volunteers, religious leaders, traditional healers, community awareness, social interaction, family and peer support)

a. Who has supported you in accessing palliative care (family, friends, healthcare providers)?

17. **What factors do you believe enable effective palliative care for female cancer patients?**

**Probe:** Are there cultural or societal factors that impact the delivery of palliative care?

a. What resources (e.g., medical staff, facilities) are available to you for palliative care? Are there specific areas where you believe improvements can be made?
b. How do you perceive the quality of palliative care services which you receiving?

18. **How have financial factors influenced your access to palliative care?**

**Probe:** Are there any financial assistance programs available that you are aware of?

**• In your opinion, what changes could be made to improve access to palliative care for female cancer patients?**

**Probe:** What additional support do you think would help women like yourself in accessing comprehensive palliative care?

F. **Gaps/Challenges**
19. **What are the challenges you encountered in getting palliative care?**

**Probe:** How did you cope up?

a. Any logistical issues (e.g., transportation, availability of services)?

20. **In your opinion, what are the main gaps in the palliative care services available to you?** **Probe:** Any missing support during your treatment?
    a. Are there specific barriers related to gender that you have encountered?
21. **What is your opinion on the referral path**

**Probe:** what are the challenges to get continues care at home or community and the linkage with facility or health care providers? Did you get pain killer frequently

- Your suggestion to improve the care continuity?

22. **Based on your experiences, what suggestions do you have for improving palliative care services for female cancer patients?**

**Probe:** If you could change one thing about the palliative care, what would it be?

a. Is there anything else you would like to share about your experiences with palliative care?

**Objective**

- To explore palliative care needs of female cancer patients, and their caregivers in Ethiopia
- To explore enablers, and challenges to improve the continuum of care through comprehensive palliative care model for female cancer patients in Ethiopia

2. **For Care givers**
A. **Socio-demographic and Clinical Characteristics**
1. **Can you tell me about yourself?**

**Probe:** age, sex, educational level, marital status, job, source of income, family size and living conditions?

- General health status

B. **Understanding the Caregiving Experience**
2. **Are you familiar with palliative care? If yes, how would you describe it?**

**Probe:** Have you or the person you care for received any palliative care services? If so, what was your experience like?

- How long have you been serving as a caregiver? your relationship to the person you are caring for?

3. **Can you describe your daily responsibilities as a caregiver?**

**Probe:** What types of care do you provide (e.g., physical, psychosocial, spiritual, economical, end of life care)?

a. What are some of the biggest challenges you face in your role?
b. How do you manage your own physical and emotional well-being while caring for someone else?

C. **Palliative care Needs**
4. **How do you prioritize the different aspects of care (physical, psychosocial, spiritual, economical, end of life care)?**

**Probe:** What do you believe are the most important aspects of palliative care for caregivers and patients?

a. What specific needs do you have as a caregiver in terms of emotional, physical, social, economic, end of life care?

5. **What resources or services do you think would help improve your caregiving experience?**

**Probe:** Are there community resources available to you? If so, how effective do you find them?

6. **How does your cultural background influence your approach to caregiving and palliative care?**

**Probe:** Are there traditional practices or beliefs that affect how you provide care?

- How do family dynamics play a role in caregiving within your community?

D. **Preference on Palliative care delivery approaches/models**
7. **How would you define home-based care, community-based care, and facility-based care?**

**Probe:** What are your initial thoughts about each type of care?

8. **How did healthcare providers, family, or community members support you during your palliative care journey**?

**Probe:** Were there any particular programs or services that stood out to you as beneficial?

9. **Which type of care setting do you prefer (home-based, community-based, or facility-based or else)? Why? (**Care provider, timing, required materials, why?)
   a. What factors influence your preference for one type of care over the others?
   b. Can you describe any past experiences that have shaped your preferences for care settings?
10. **What do you see as the benefits of receiving care at home?**

**Probe:** Are there any concerns or challenges you associate with home-based care?

a. What types of services or support would you need to feel comfortable receiving care at home?

11. **What do you think are the advantages of community-based care?**

**Probe:** Have you accessed any community resources or services?

- If so, what was your experience like?
- Are there any barriers that prevent you from utilizing community-based care?

12. **What do you perceive as the benefits of receiving care in a facility**?

**Probe:** Can you share any concerns or drawbacks you associate with facility-based care?

- Under what circumstances would you consider facility-based care to be necessary or preferable?

13. **What role do your family and friends play in your decision-making regarding care options?** **Probe:** How important is the availability of healthcare professionals when considering your preferred care setting?
    a. Are there any specific services or amenities that would influence your choice of care setting?
14. **What changes would you like to see in the way care is provided across home-based, community-based, and facility-based settings?**

**Probe:** If you could design an ideal care model that incorporates elements from all three

**Probe:** What components are essential for a comprehensive care model that includes physical, psychosocial, spiritual, economic, and end-of-life care?

15. **Is there anything else you would like to share regarding your needs or experiences related to palliative care?**

**Probe:** How can caregivers, volunteers, and healthcare providers better support patients and families during this time?

- **What changes could be made to better support caregivers like yourself?**

**Probe:** What advice would you give to healthcare providers regarding supporting caregivers?

E. **Opportunities and Barriers for Comprehensive and Continuous Care**
16. **What resources or support systems do you have in place that facilitate continuous care at home or in the community?**

**Probe:** Any positive experiences or successful strategies you've used to provide comprehensive care?

17. **How do community resources (e.g., support groups, Ekub, Idir, healthcare services) assist you in your caregiving role?**
18. **What opportunities exist to enhance palliative care services? (e.g., training caregivers, community leaders, community volunteers, religious leaders, traditional healers, community awareness, social interaction at the community, family and peer support)**
19. **What factors do you believe enable effective palliative care for female cancer patients?**
20. **Are there cultural or societal factors that impact the delivery of palliative care? Are there specific areas where you believe improvements can be made?**
21. **What challenges do you encounter when trying to provide holistic and continuous care at home or in the community?**

**Probe:** Physical barriers (e.g., lack of equipment, space)

• Psychosocial barriers (e.g., stress, emotional burden)

• Spiritual barriers (e.g., lack of support for spiritual needs)

- Economic barriers (e.g., costs of care, financial strain)
- End of life care (e.g., lack of support for end-of-life needs, lack of awareness, not preferred by the patients or care givers)
- How do these barriers impact the quality of care? And how do you address these barriers?
- Are there specific situations or events that highlight these challenges?

22. **What costs are associated with caregiving that you did not anticipate?**

**Probe:** Are there any financial assistance programs or resources available to you? How effective are they?

- How does the financial situation of the person you are caring for affect your ability to provide care?

23. **How do you approach end-of-life care for the person you are caring for?**

**Probe:** What support do you feel is necessary for caregivers during this stage?

- Are there cultural or spiritual beliefs that influence how end-of-life care is provided?

F. **Communication and Ethics**
24. **What dilemmas have you faced while providing palliative care for cancer patients?**

**Probe:** Can you share a specific situation where you felt conflicted about a decision related to care?

- What were the factors influencing your decision?
- How do you balance the wishes of the patient with those of other family members or healthcare providers?

25. **How do you approach discussions about prognosis and treatment options with the patient and their family?**

**Probe:** What challenges do you face in ensuring that patients understand their options and make informed decisions?

- Are there instances where you felt that informed consent was not adequately obtained? How did you handle those situations?
- Have there been times when a patient's wishes conflicted with what you believed was in their best interest? How did you navigate that situation?
- What strategies do you use to advocate for the patient's preferences and values?
- How do you respect the autonomy of the patient while providing care?

26. **What support systems do you rely on when faced with dilemmas in palliative care?**

**Probe:** Are there resources (e.g., community advisory board/committee counseling) that you have found helpful in addressing these issues?

a. How can healthcare institutions better support caregivers in managing ethical challenges?

27. **What changes would you suggest to improve decision-making processes in palliative care settings?**

**Probe:** How can training or education for caregivers be enhanced to address care issues more effectively?

**• Is there anything else you would like to share regarding your experiences with ethical issues in palliative care?**

**Thank you!**

**Objective**

- To explore enablers, and challenges to improve the continuum of care through comprehensive palliative care model for female cancer patients in Ethiopia

3. **For rebellious and community leaders**
28. **Can you tell me about yourself?**

**Probe:** age, sex, educational level, marital status, job, source of income, family size and living conditions?

29. **Are you familiar with palliative care? If yes, how would you describe it?**

**Probe:** Have you or the person you care for received any palliative care services? If so, what was your experience like? In your opinion what is the components of palliative care?

30. **How are you involved in supporting patients**

**Probe:** How long have you been serving as a religiouse/community leader? your relationship to the person you are supporting for?

31. **Can you describe your daily responsibilities as religiouse/community leader?**

**Probe:** What types of care do you provide (e.g., physical, psychosocial, spiritual, economical, end of life care)? What are some of the biggest challenges you face in your role? How do you manage your own physical and emotional well-being while caring for someone else?

32. **What services are provided in your community to severely ill/cancer patients?**

**Probe:** Who is providing these services? What training did they receive? *by whom training was provided?*

33. **What kind of services are providing for patients?**

**Probe:** Spiritual care, Pyscho-social, Economic, indigenous medicine, who is providing these services? How, with practical example? Home based; community based? Based on what criteria are patients included in the program? What are the preferences or needs of the patients? Who can access these services?

34. **How are family members of severely ill/cancer patients supported?**

**Probe:** How does the community view severely ill patients? How does the community approach family of severely ill patients?

35. **Do you work together with other organizations or programs?**

***Probe:*** *If yes, whom?* What are the strong points of the program? What would you like to improve about the program? Do you work together with other health care providers? *If yes, who?*

36. **What are the existing opportunities to improve the continuum of care?**

***Probe:*** What is locally available resource? Traditional healers, spiritual care, social gatherings and supports like Equb/Idir

37. **What are the existing opportunities to provide comprehensive palliative care?**

**Probe:** What are then enablers to optimize comprehensive palliative care delivery, Service integration, Traditional healers, spiritual care, social gatherings and supports like Equb/Idir

38. **What are the existing opportunities to provide community-based PC, home based care?**
39. **What training did the community representative/traditional healers/religious leaders receive?**

***Probe:*** *by whom training was provided*

40. **How would you like to improve your contribution for supporting cancer patients?**

**Probe:** What is your need to improve your service?

41. **What are barriers for patients to access palliative care?**

**Probe:** What are the challenges/barriers that affect the continuum of care, what are the challenges/barriers that affect the delivery of comprehensive PC, what are the hinder factors to have integrated services at all levels, Community awareness, misconception, norm/culture/religion beliefs???

42. **What are current gaps you experience in palliative care?**

**Probe:** What are priorities for improving palliative care? Who should be responsible to work on these priorities?

43. **What are your expectations for palliative care in the future?**

**Probe:** What is your suggestion to improve the delivery of palliative care?

44. Do you have any other comments?
45. Do you have any questions?

***Thank you for your time and participation!***

**Objective**

- To explore enablers, and challenges to improve the continuum of care through comprehensive palliative care model for female cancer patients in Ethiopia

4. **Healthcare providers, and key informants**
46. **Can you tell me about yourself?**

**Probe:** age, sex, educational level, marital status, job, source of income, family size and living conditions?

**PC experience**

47. **Can you describe what palliative care is, in your own words?**

**Probe:** In your opinion what are the components of palliative care? What is comprehensive palliative care delivery?

48. **Are you aware of the national protocol on palliative care?**

**Probe:** If yes, which version?what are the content of the guideline? What is the strength of the guideline, what is lacking?

49. **How are you involved in palliative care?**

**Probe:** Do you work together with other health care providers? *If yes, with whom?*

50. **How is your organization involved in palliative care?**

**Probe:** What are the services currently provided? Who is providing these services?

**Enablers to comprehensive and continues care**

51. **What are the existing opportunities to improve palliative care**?

**Probe:** What are the existing opportunities to improve the continuum of care

- What are the existing opportunities to improve referral path from facility to home, from home to facility
- what are the existing opportunities to provide comprehensive care

52. **What is locally available resource which enhance comprehensive palliative care delivery?**

**Probe:** service integration, community-based PC, home based care, traditional healers/ indigenous medicine volunteers, social gathering like Idir/Equb….

53. **What training did the providers receive?**

**Probe:** When, by whom training was provided, what was the content of the training

54. **Do you work together with other organizations or programs**?

**Probe**: Based on what criteria are patients included in the program?

- Who can refer to the program?
- What are the strong points of the program? What would you like to improve about the program?

55. **What services are provided in your community to severely ill patients?**

**Probe:** Who is providing these services?

- What training did the providers receive?
- By whom training was provided?
- Who can access these services?

56. **How are family members of severely ill patients supported?**

**Probe:** How does the community view severely ill patients?

- How does the community approach family of severely ill patients?

**Challenges to comprehensive and continues care**

57. **What are current gaps you experience in palliative care?**

**Probe:** What are barriers for patients to access palliative care?

- What are the challenges/barriers that affect the continuum of care?
- What are the challenges/barriers that affect the delivery of comprehensive PC
- What are the hindering factors to have integrated services at all levels

58. **What are priorities for improving palliative care?**

**Probe:** What would you like to improve about the program?

Who should be responsible to work on these priorities?

59. **What are your expectations for palliative care in the future?**

**Probe:** For the health care providers?

60. **Do you have any other comments/any questions?**

***Thank you for your time and participation!***
